# Analytical Formalism for Data Representation and Object Detection with 2D LiDAR: Application in Mobile Robotics

**DOI:** 10.3390/s24072284

**Published:** 2024-04-03

**Authors:** Leonardo A. Fagundes, Alexandre G. Caldeira, Matheus B. Quemelli, Felipe N. Martins, Alexandre S. Brandão

**Affiliations:** 1 Robotics Specialization Center (NERo), Department of Electrical Engineering, Federal University of Viçosa, Viçosa 36570-000, MG, Brazilalexandre.caldeira@ufv.br (A.G.C.); matheus.quemelli@gmail.com (M.B.Q.); 2 Graduate Program in Computer Science, Department of Informatics, Federal University of Viçosa, Viçosa 36570-000, MG, Brazil; 3 Sensors and Smart Systems Group, Institute of Engineering, Hanze University of Applied Sciences, 9747 AS Groningen, The Netherlands; fe.nascimento.martins@pl.hanze.nl

**Keywords:** LASER scanner, LiDAR, object detection, object localization, mobile robotics

## Abstract

In mobile robotics, LASER scanners have a wide spectrum of indoor and outdoor applications, both in structured and unstructured environments, due to their accuracy and precision. Most works that use this sensor have their own data representation and their own case-specific modeling strategies, and no common formalism is adopted. To address this issue, this manuscript presents an analytical approach for the identification and localization of objects using 2D LiDARs. Our main contribution lies in formally defining LASER sensor measurements and their representation, the identification of objects, their main properties, and their location in a scene. We validate our proposal with experiments in generic semi-structured environments common in autonomous navigation, and we demonstrate its feasibility in multiple object detection and identification, strictly following its analytical representation. Finally, our proposal further encourages and facilitates the design, modeling, and implementation of other applications that use LASER scanners as a distance sensor.

## 1. Introduction

LiDAR, short for Light Detection and Ranging, is a crucial tool in instrumentation and robotic navigation. Its primary advantage lies in its ability to accurately calculate the depth, shape, and dimensions of objects based on captured data [1]. Nowadays, these devices play a key role in assisting robots in performing autonomous navigation tasks in indoor environments, such as factory corridors and warehouses [2]. Also known as LASER sensors, LiDARs are particularly suitable for capturing time series data [3], generating point clouds [4,5], and acquiring regular angular depth data [6].

**Figure 1:**
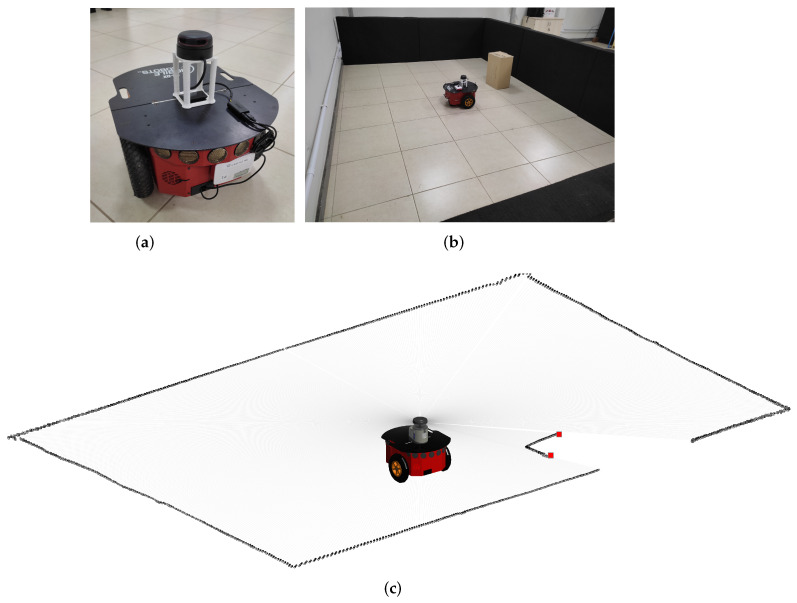
(**a**) Robot with a 2D LiDAR sensor mounted on its top (on the white frame). (**b**) View of the robot in its environment with one static object in front of it. (**c**) Resulting 2D LiDAR measurements—the edges of the static object are highlighted by red squares.

LiDARs can be 1D, 2D or 3D: 1D LiDAR, commonly known as a laser range finder (LRF), is the core of the LiDAR scanner system, while a 2D LiDAR employs a LASER beam to gauge distances from the sensor to objects within a plane surrounding it. A 3D LiDAR operates in a similar way, but measures distances from the sensor to objects within a surrounding sphere around the sensor [7]. Figure 1 illustrates the use of a 2D LiDAR and its measurements. The robot depicted in Figure 1a is equipped with an omnidirectional 2D LiDAR sensor mounted on its top. Figure 1b shows the robot inside a rectangular area sorrounded by walls, with a static object (a box) in front of it. Corresponding LiDAR measurements are depicted in Figure 1c, where the edges of the box are highlighted by red dots.

A notable example of a widely applied technique that uses LASER scanners is Simultaneous Localization and Mapping (SLAM), the procedure of autonomously building a map while a robot is localizing itself in the environment [8]. Research related to this topic within the field of mobile robotics has remained popular for a long time, and more recently, additional efforts have been made to contribute to the development of intelligent and autonomous vehicles [9,10], a field in which many works focus on object detection methods using 3D LiDAR [11,12,13]. On the other hand, 2D LiDARs are preferred in many mobile robotics applications due to their low cost and high degree of accuracy, since it is well suited for flat, indoor spaces [14]. The application also plays a role in the choice of sensor. For example, in places such as electrical substations, optical sensors are preferred to obtain distance information because they do not suffer interference from large electromagnetic fields [15].

Besides the approaches mentioned above, there are many others that motivate and drive the purpose of this work, in particular the use of LASER scanners for object detection and tracking (including cases in which both the agent and objects are mobile) [16,17,18,19], object identification and segmentation from the local environment [20,21,22], and object feature extraction [23]. These implementations have a deep impact on autonomous robotics and decision making, using little or no prior knowledge about the environment and objects yet accurately inferring information and executing tasks based on such data.

In yet another similar sense, SLAM implementations frequently focus on building and self-correcting a map or a CAD (Computer-aided design) model map based on LASER scanner data. Generally, many such techniques apply triangulation, environment landmarks [5], and object feature detection [24] for systematic odometry error compensation in both indoor [25,26,27] and outdoor [28,29] data. In cases where a map is already available, the use of 2D LiDAR is also attractive. For instance, a fast obstacle detection technique for mobile robot navigation using a 2D-LiDAR scan and a 2D map is proposed in [30].

Nonetheless, other fields also benefit from the use of LASER scanners. In the agricultural automation industry, for example, there are various research efforts in the evaluation of canopy volume [31], poplar biomass [32], trunks [33], and crop and weed distinction [34], among other uses. From a different perspective, the robotics competition RoboCup and its educational counterpart RoboCupJunior, specifically in the Rescue [35] and Rescue B [36] categories, respectively, have also benefited from using LASER range data for robot navigation in unstructured environments to perform rescue operations.

Thus, there is extensive literature on 2D LiDAR data applications in detecting, locating, and matching objects, as well as in map construction, matching, and correction for self-localization. However, to the best of our knowledge, there is no clear universal consensus on strict mathematical notation and modeling for such instruments, although they have lower computational cost than image recognition processes [18]. Wang et al. [37] also state that there is a need for standardization of information extraction based on LiDAR data. They propose a framework from semantic segmentation to geometric information extraction and digital modeling, but their focus is on the extraction of geometric information from roads.

In order to process LASER scan information, each paper in the literature suggests its own notation, framework, and approach. This lack of standardization is sub-optimal for scientific research, development, and education, where a unified approach would be preferable. Considering all aforementioned applications, we contend that it is valuable and significant to propose and evaluate a formal mathematical definition for object detection and identification in tasks based on segmentation, tracking, and feature extraction.

Given the wide array of applications based on and benefiting from LiDAR data, there is as of yet no rigid definition or analytical approach for the general problem of detecting objects in semi-structured environments. In other words, despite the existence of similar structures, there is a gap between different approaches.

### 1.1. Related Works

There is a multitude of applications and strategies already proposed and validated in the literature for the use of LiDAR sensors in autonomous navigation, each with its own specific advantages and disadvantages. The versatility and cost effectiveness of LiDAR-based distance measurement instruments have spurred rapid advances in robotics. Their ability to detect and identify objects, obstacles, other robots, and humans within a scene significantly impacts the planning algorithms of autonomous robots. In this sub-section, we list some relevant work that illustrates how LASER sensor data are used and represented in different applications. In the following subsection, we explain how our proposal contributes to the state of the art.

In the literature, it is common to find a correspondence between the representation of Cartesian space measurements for various mapping and terrain reconstruction applications, such as forest areas, highway structures, and power transmission lines [38,39,40,41]. In those applications, the measurement point is associated with a reference, whether fixed or mobile.

In [1], sensor measurements can be regarded as a representation of points in space, where each point corresponds to the reflection of the beam in the environment. In other words, measurements are recorded as a point cloud [42], which can later be associated with object detection and evasion strategies or for environment location.

Robot navigation relies on sensor influx and fusion to extract environmental features and deliberate upon its surroundings to execute a task, whether simple or complex. In that sense, LiDAR sensors are widely used in SLAM and often depend on feature mapping and tracking to achieve precision and accuracy using deterministic and probabilistic models, as seen in the literature [5,8,26,27]. Similar techniques are also used in the research of autonomous driving [9,10].

A strategy for detecting circular objects based on their geometric properties and polar curve fitting is presented in [43]. To enhance this technique, support vector machines (SVMs) are employed for object detection, proving applicable to robot localization and navigation tasks. In line with this, to improve the SLAM process, the Polar Scan Matching (PSM) strategy proposed in [44] demonstrates efficiency in associating points based on bearing similarity, allowing faster processing compared to traditional iterative methods, such as Iterative Closest Point (ICP). Furthermore, in [45], the representation of angular measurements as a Gaussian process during the matching stage improves navigation map representations compared to ICP and PSM.

LiDAR sensors are also used to detect and track objects from consecutive distance measurements to predict cluster routes by particle filter algorithms [46] or by Kalman Filters after a hierarchical object association [47]. Similarly, a tracking technique is employed in [48] to follow objects in port terminals using autonomous guided vehicles (AGVs) without prior information about shape or size. To avoid obstacles in unknown environments, LASER sensors infer the location of possible collision points and maneuver to avoid them [49,50]. Furthermore, in relation to navigation, measurements can be used to detect and track moving objects or to infer the width of the corridor, allowing or not allowing autonomous agents to pass [51].

The detection and identification of objects and their properties is imperative for navigation and task completion in mobile robotics. A standard mathematical framework to interpret LASER data can be fruitful to describe as well as improve models and implementations, e.g., in the field of forest and agriculture robotics applications [31,32,33,34]. Thus, formal investigations and modeling of the physical world for autonomous interpretation by robots is impactful.

In contrast with the works cited above, the present proposal considers LASER scanning as a function that relates distance information given the measurement angle. To translate the result of this discrete function into a point cloud, the temporal history of the measurements must be obtained. Thus, the proposed representation can become equivalent to those presented in other works in the literature.

### 1.2. Contributions and Organization of the Article

A precise mathematical formulation for object detection and identification, particularly in tasks involving segmentation, tracking, and feature extraction, holds significant value across various applications in both research and industry. In light of this, our primary contribution is the formal definition of LASER sensor measurements and their representation. This encompasses not only the identification of objects but also the delineation of their key properties and spatial locations within a scene. We achieve this by uniquely representing each object through mathematical notations, explicitly situating them within the set of objects that collectively constitutes the entire universe set. Here, the universe set denotes the comprehensive environment enveloping an agent.

In essence, this paper tackles the formalization of distance measurement and object detection using LASER sweep sensors, specifically 2D LiDARs. The application of this framework is then discussed, encompassing aspects of object detection, localization, and matching within a broader context. The paper begins by presenting an overview of the problem, discussing related formalization efforts, and highlighting works that stand to gain from a standardized modeling framework. Subsequently, our contribution unfolds across three main sections of theoretical modeling, followed by experimental validation with a real robot. Initially, the scope is defined, outlining how LiDAR scan measurements can be mathematically represented. Following this, the framework is employed to deduce properties from objects within a scene. Finally, a comprehensive guideline for object detection and localization is established through practical application, shedding light on the advantages of our proposed modeling approach in a realistic semi-structured environment. The ultimate objective is to facilitate accessible and universally applicable research, exploring the merits and potential limitations of LiDAR sensors across diverse realms of robotics, be it educational, theoretical, or applied.

The subsequent sections of the article are structured as follows. Section 2 introduces the primary contribution of this work, which revolves around the proposed formalism for object identification and localization based on the sweep readings of a 2D LiDAR. The section commences by elucidating the measurements obtained from each laser beam, followed by the representation of the entire set as a discrete function that correlates the measurement angle with the recorded distance. A detailed presentation of the formalism for deducing the detection and location of objects through an analytical depiction of this function ensues. Moving forward, Section 3 encompasses the experimental and numerical validation results of our proposal, conducted in didactic scenarios with straightforward configurations. Finally, Section 4 outlines the key conclusions drawn from the study and suggests potential directions for implementing the proposed formalism in autonomous navigation tasks.

## 2. Proposed Formalism for Object Identification and Localization

In the realm of robotics applications, the characterization of a navigation environment hinges on the quantity and arrangement of objects within the scene, coupled with the degree of freedom afforded to the agents. Within this framework, an environment earns the label “structured” when the agent undertaking tasks possesses prior knowledge of the spatial arrangement of objects, and these objects either remain static or undergo changes that are entirely anticipated during task execution. Conversely, if objects exhibit unpredictable movement while the agent is in the process of executing tasks, the environment is deemed “unstructured”. Lastly, environments where a certain level of object mobility is acceptable, prevalent in settings like offices, laboratories, residences, storage facilities, and workshops, fall under the classification of “semi-structured environments”.

In the context of semi-structured environments, the navigation scene’s entities can be effectively mapped by an agent utilizing a distance sensor, which, in the scope of this study, is specifically addressed as a 2D LiDAR LASER scanner. These entities encompass both fixed objects such as walls, shelves, and wardrobes, as well as dynamic objects like boxes or other mobile agents.

### 2.1. Representation of 2D LiDAR Sweeps

The 2D LiDAR employs a LASER beam to gauge distances from the sensor to objects within a plane surrounding it. Typically in mobile robotics applications, the LASER beam rotates parallel to the ground, providing the robot with crucial information about its proximity to obstacles in its vicinity. It is essential to consider the varying ranges and resolutions of different sensors.

**Figure 2:**
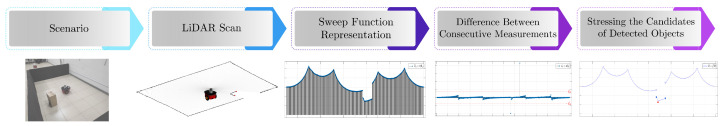
Pipeline for the proposed formalism. From left to right: the scenario under consideration, the corresponding LiDAR measurements, the sweep function corresponding to the LiDAR scan (distance versus measurement angle), the difference between consecutive distance measurements, and the identification of candidates for potential objects. The latter three figures are explained in greater detail in the following sections.

To better understand the forthcoming definitions, we consider the pipeline depicted by Figure 2, in which a robot with a 2D LiDAR mounted on its top is placed on a specific scenario (to facilitate understanding, the scenario is the same one illustrated in Figure 1). In Figure 2, the leftmost image represents the scenario under consideration. The subsequent image delineates the LiDAR measurements corresponding to the aforementioned scenario. Subsequently, the LiDAR scan undergoes processing to derive the sweep function, calculate the difference between consecutive distance measurements, and identify potential candidates for detected objects.

In the definitions given in the sequence, the subscript *k* designates a discrete set of elements (number of measurements per revolution of the LASER), and *n* denotes an element within such a set, both inherently discrete in nature.

**Definition 1.**  *We let r be a discrete function representing a LiDAR sensor, denoted by*

$$\begin{array}{cc} r:\Theta_{k} & \to D_{k}\\ \theta_{n} & \mapsto D_{n}=r\left(\theta_{n}\right), \end{array}$$

**Figure 3:**
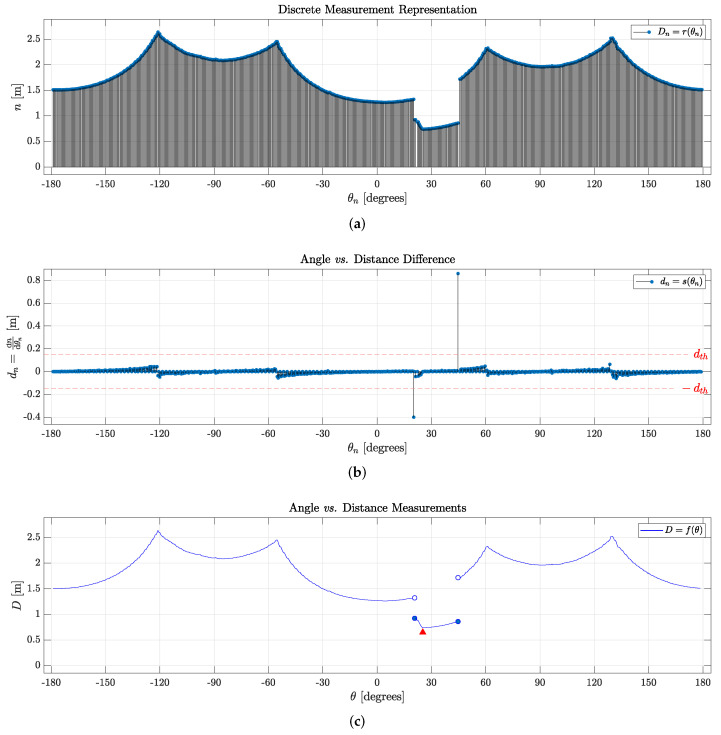
Polar stem plots representing one sweep of a 2D LiDAR scan corresponding to the scenario depicted in Figure 1: (**a**) Discrete distance measurements per sample; (**b**) Distance difference between subsequent samples; (**c**) Contour given by the distance measurements. In all cases, the angle represents the orientation of the LASER beam with respect to the base of the sensor, which has a range of 360°.

*where domain $\Theta_{k}$ indicates a set containing each discrete angle within the angular scan range, and co-domain $D_{k}$ denotes the set of measurements assigned to each angle $\theta_{k}$. Such a discrete function is shown in Figure 3a.*

**Definition 2.**  *We let s be a difference function given by*

$$\begin{array}{cc} s:\Theta_{k} & \to d_{k}\\ \theta_{n} & \mapsto d_{n}=r\left(\theta_{n}\right)-r\left(\theta_{n-1}\right)=s\left(\theta_{n}\right), \end{array}$$

*where $\theta_{n}$ is an element in the set $\Theta_{k}$ of all angles within the angular range of the instrument, and $d_{k}$ is a set of differences between two neighboring consecutive measurements, as shown in Figure 3b.*

**Definition 3.**  *We let f be a function coinciding with r($\theta_{n}$) $\forall \theta_{n}\in \Theta_{k}$, i.e.,*

$$\begin{array}{cc} f:R & \to R\\ \theta & \mapsto D=r(\theta_{n}), \end{array}$$

*such that f is also continuous and monotonic in intervals $\left(\theta_{n-1},\theta_{n}\right)$ for every n=1,⋯,N (see Figure 3c), whose one-sided limits are*

$$\begin{array}{cc} \lim_{\theta \to \theta_{n}^{-}}f\left(\theta \right) & =f(\theta_{n-1}),\\ \lim_{\theta \to \theta_{n}^{+}}f\left(\theta \right) & =f(\theta_{n+1}), \end{array}$$

*whenever $|s\left(\theta_{n}\right)|>d_{th}$. Here, $N=card(\Theta_{k})$ denotes the set cardinality of $\Theta_{k}$ (representing the sensor’s resolution) and $d_{th}$ is a case-specific threshold value, serving as a free parameter that represents the minimal difference in distance measurements for object detection. To automate the object detection and identification process, a metric for $d_{th}$ can be computed as the mean absolute difference value*

$$\begin{array}{c} d_{th}=\sum_{n=1}^{N}\frac{|d_{n}\left(\theta_{n}\right)|}{N}, \end{array}$$

*to separate noise from actual meaningful data, as discussed further. An illustrative example is presented in a figure in Section 3.*

**Proposition 1.**  *For a properly functioning 2D LiDAR sensor, $\forall \theta_{n}\in \Theta_{k},\;\exists r\left(\theta_{n}\right)|D_{n}=r\left(\theta_{n}\right)$.*

**Proof.**  The LiDAR sensor assigns a distance measurement reading for each angle within its operational range, assuming the sensor functions correctly and is free from manufacturing errors. Any malfunctions or manufacturing errors should be identified and rectified through appropriate assessment and correction procedures. □

**Corollary 1.**  *If Proposition 1 is fulfilled, the mapping $r:\theta_{n}\to D_{k}$ is, by definition, surjective.*

**Corollary 2.**  *Proposition 1 and Corollary 1 imply that f is surjective by definition, as r aligns with f.*

In the preceding text, Definition 1 delineates how the agent perceives its navigation surroundings. It is noteworthy that, as inferred from Corollary 2 and Definition 3, *f* is differentiable across a significant portion of its domain. Points where *f* lacks differentiability hold crucial significance, especially in the context of defining objects within a LASER’s scan data. It is essential to highlight that for all θ in $\left[\theta_{\min},\theta_{\max}\right]$ and all *D* in $\left[0,D_{\max}\right]$, their extreme values are contingent upon the model and manufacturer specifications of the sensor device.

### 2.2. Definition of Objects

Initially, we establish U as a set of points symbolizing the entire environment from the robot’s perspective, consisting of distinct sets: objects, agents (comprising both humans and robots within the environment), and additional task-unrelated data, regarded as noise. It is evident that these three constituent sets forming U are disjoint among each other.

**Definition 4.**  *We let U represent a universe set, populated by LiDAR measurements and exclusively composed of a set of objects O, a set of agents A, and a set of noise S. Consequently,*

$$\begin{array}{c} U=\{A\cup S\cup O:O\cap A=O\cap S=A\cap S=O\cap A\cap S=\emptyset \}. \end{array}$$

Given that *f* is a continuous function, its differentiability may vary. However, if *f* is differentiable at point *a*, then *f* is not only continuous at *a* but also laterally continuous: $f_{-}^{\prime }\left(a\right)=f_{+}^{\prime }\left(a\right)$. In simpler terms, the left-hand and right-hand derivatives at *a* must exist and possess identical values. Leveraging the concept of differentiability allows for the discernment of objects, walls, and free space in a LiDAR scanner reading. Specifically, it follows that if there exists a point where f(θ) is not differentiable and that point falls outside the interval of an object, it must be the edge of a wall (a corner); otherwise, the point belongs to the edge of an object. This concept is illustrated in Figure 3c: the discontinuities in $\theta =26^{{}^{\circ}}$ and $\theta =35^{{}^{\circ}}$ represent edges of the box in front of the robot (as shown in Figure 1).

**Definition 5.**  *We consider any prismatic object O within a semi-structured environment. Then, O can be described as a set of points in polar coordinates:*

$$\begin{array}{c} O=\left\{\left(\theta ,r(\theta )\right)\in R^{2}/\theta_{i}\leq \theta \leq \theta_{f}\right\},\\ \forall \theta_{i,f}/f_{-}^{\prime }\left(\theta_{i,f}\right)=f_{+}^{\prime }\left(\theta_{i,f}\right), \end{array}$$

*where $\theta_{i}<\theta_{f}$, with $P_{i}=\left(\theta_{i},r\left(\theta_{i}\right)\right)$ representing a point of discontinuity and $P_{f}=\left(\theta_{f},r\left(\theta_{f}\right)\right)$ being the immediately succeeding point of discontinuity to the right of $P_{i}$. These points collectively define the starting and ending measurements of an object’s body. Consequently, f(θ) maintains continuity within open interval $\left(\theta_{i},\theta_{f}\right)$.*

It is important to note that O is defined as prismatic to facilitate the definition of faces and vertices. We consider a generic prismatic object along with its corresponding polar coordinates contained within O. It is noteworthy that within any such set O, a discontinuity in the derivative of f(θ) signifies an edge, denoted by red triangles in Figure 3c. Consequently, it becomes possible to define both the faces and vertices that pertain to O.

**Definition 6.**  *We let V be a set of points representing any edge of any prismatic object such that*

$$\begin{array}{c} V_{k}=\left\{\left(\theta ,r(\theta )\right)\in R^{2}/f^{\prime }\left(\theta \right)=0,\theta \in O\right\},\;\mathrm{with}\;k=0,1,2,\cdots \;,n. \end{array}$$

**Definition 7.**  *We consider O as any prismatic object, and we let $F_{k}$ denote a set of points representing the kth face of this object. In polar coordinates, this can be defined as*

$$\begin{array}{c} F_{k}=\left\{\left(\theta ,r(\theta )\right)\in R^{2}/\theta_{k}\leq \theta \leq \theta_{k+1}\right\},\;\mathrm{with}\;k=0,1,2,\cdots \;,k, \end{array}$$

*where $\theta_{0}=\theta_{i}$, $\theta_{n}=\theta_{f}$ and all $V_{k}$ are in $\left(\theta_{k},f\left(\theta_{k}\right)\right)$.*

In simpler terms, in accordance with Definition 7, the edges of the object are situated at a local minimum or maximum between two faces based on LASER’s readings. Furthermore, all faces are located within interval $\left(\theta_{i},\theta_{f}\right)$, leading to inclusion relation $O\subseteq V_{k}\cup F_{k}$.

Hence, referring to Figure 3c, function f(θ) exhibits discontinuities at $\theta_{1}$ and $\theta_{2}$. Consequently, it becomes plausible to assert that every element $\theta \in [\theta_{1},\theta_{2}]$ corresponds to a measurement from the surface of an object, thereby establishing all essential conditions to infer the existence of an object. It is important to note that O is defined as prismatic to facilitate the definition of faces and vertices. However, the same discontinuity-based definition can be applied to identify other, perhaps more irregularly shaped objects.

The presented methodology could be applied in many works, serving as a guide for LASER sweep representation, highlighting regions of interest in data, and establishing standardized notation. To further illustrate and validate the reliability of this strategy, generic representative cases are presented in the following section.

## 3. Detection and Localization Experiments

This section elucidates the behavior of the 2D LiDAR sensor in a real-world environment and delineates how the formalism proposed in this article is applied to represent the scene and identify potential objects of interest. The robot employed in the experiments is depicted in Figure 1a. It is a Pioneer 3-DX controlled by a Raspberry Pi running RosAria, equipped with an omnidirectional 2D LiDAR sensor mounted on its top.

To demonstrate the efficacy of our proposal, we present a scenario featuring objects with diverse configurations, sizes, and shapes, aiming to construct an environment that accurately emulates a real-world use case. In this scenario, a mobile robot navigates along a super-ellipse trajectory around objects positioned in the center of the environment. Measurements from the 2D LiDAR sensor are utilized to construct views of the scene as the robot navigates. The LiDAR sensor is configured with a depth range of 0.1 to 12 m, a resolution of 361 measurements per revolution, and a sampling rate of one revolution per 100 ms. Following our notation, the LASER’s domain is $\Theta_{k}=\left[-180^{{}^{\circ}},180^{{}^{\circ}}\right]$, where $N=\mathrm{card}(\Theta_{k})=361$ (for k=1,2,⋯,361), and the codomain is D∈[0.1,12] m, in accordance with Definition 3. For guiding the navigation of Pioneer 3-DX, a previously validated controller is employed [52].

**Figure 4:**
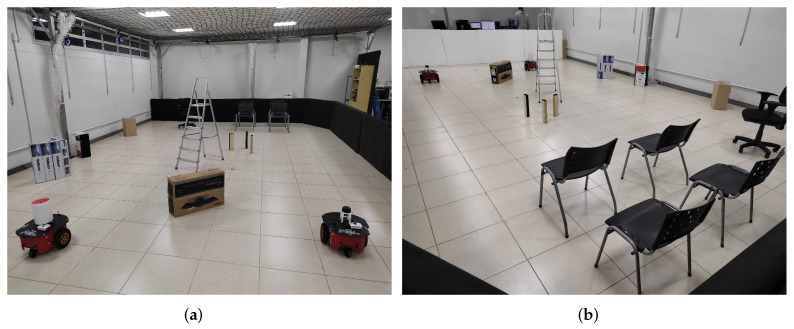

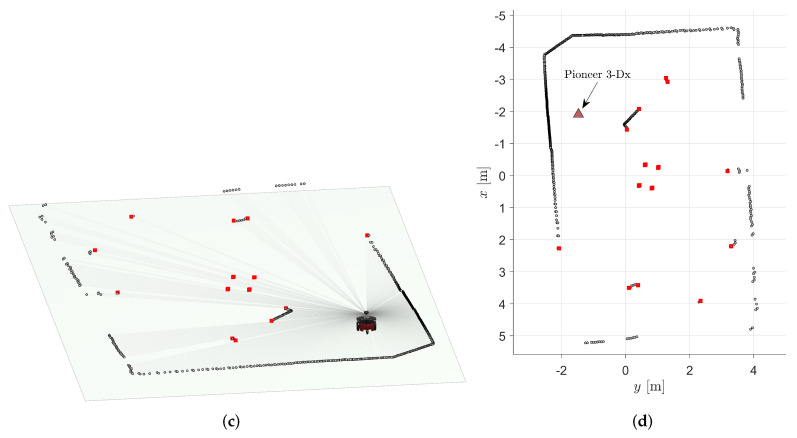
Experimental environment employed for validating the sensory mathematical representation. (**a**,**b**) are views of the same scenario in different conditions and angles. (**c**,**d**) are the 2D LiDAR readings corresponding to scenes (**a**,**b**), respectively.

Figure 4 provides a visual representation of the experimental environment employed for validating the mathematical representation of the sensor data. In the depicted views, one can observe rectangular boxes, chairs with legs and wheels, a four-legged ladder, a second mobile robot, and the surrounding walls that define the scenario. This configuration facilitates the identification of objects based on the discontinuities observed in the measurements, as conceptualized in Section 2.

**Figure 5:**
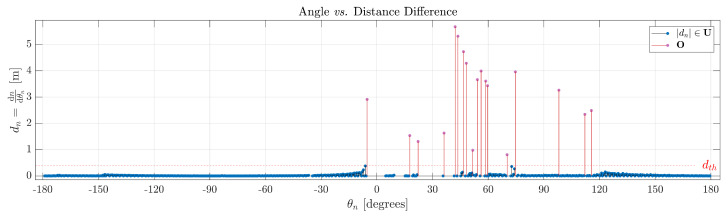
Resulting selection from Figure 4 based on $d_{th}$, according to Definition 3.

To confirm the validity of the proposed approach, utilizing Definition 3, $d_{th}$ establishes, computes, and distinguishes the objects in the scene. Figure 5 illustrates that all red lines signify a set of measurements of interest, suggesting a potential object. It is noteworthy to emphasize that the vertices of the objects, specifically their starting and ending boundaries, are derived from the difference function $s(\theta_{n})$.

**Figure 6:**
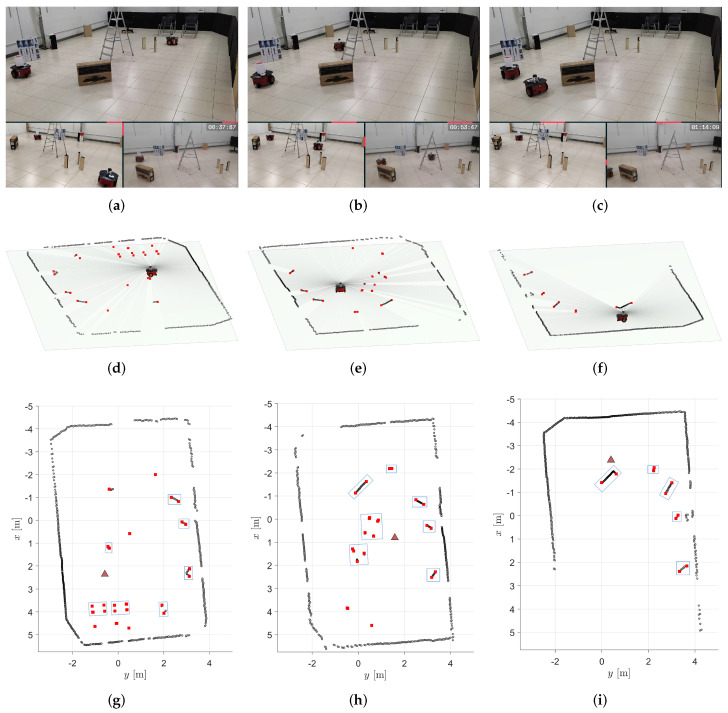
Snapshots of the validation experiment (**a**–**c**) with their corresponding 2D LiDAR readings from the robot’s perspective (**d**–**f**), along with the 2D representations in the world featuring bounding boxes of identified objects according to the proposed formalism (**g**–**i**).

**Figure 7:**
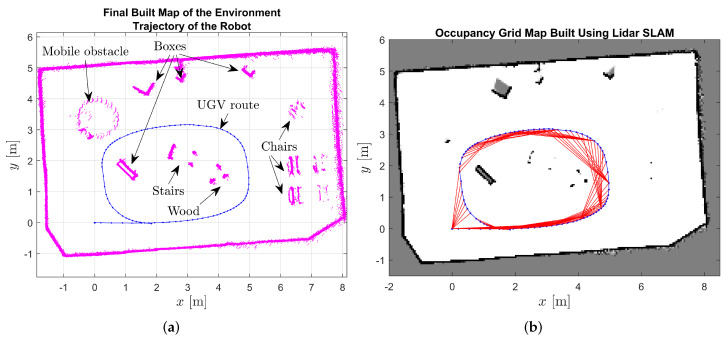
Mapping of the surroundings based on LiDAR measurements during robot displacement. (**a**) Point cloud stored during the experiment. (**b**) Occupancy grid based on laser measurements.

To exemplify the identification of objects during the robot’s navigation, the first row of Figure 6 showcases three snapshots of the robot’s path. The second row of Figure 6 displays the corresponding 2D LiDAR scans, while the third row presents the 2D reconstruction of the world from the mobile robot’s perspective, with the blue bounding boxes representing the identified objects. Video https://youtu.be/X57udApLx1w (accessed on 30 March 2024) demonstrates the execution of this experiment. Moreover, to further augment our understanding of the environment, additional visualizations are provided in Figure 7. Figure 7a shows the point cloud of measurements accumulated throughout the entire experiment, offering a comprehensive overview of the captured data. Conversely, Figure 7b presents an occupancy grid derived from laser measurements, providing a structured representation of the environment’s occupancy status. Together, these visualizations enhance our comprehension of the robot’s navigation process and its interaction with the surrounding environment.

**Figure 8:**
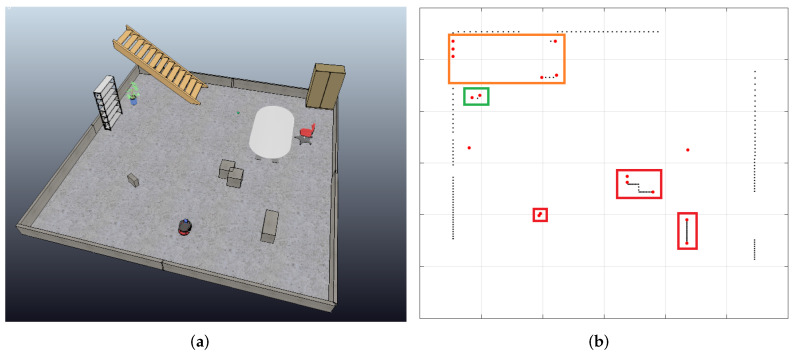

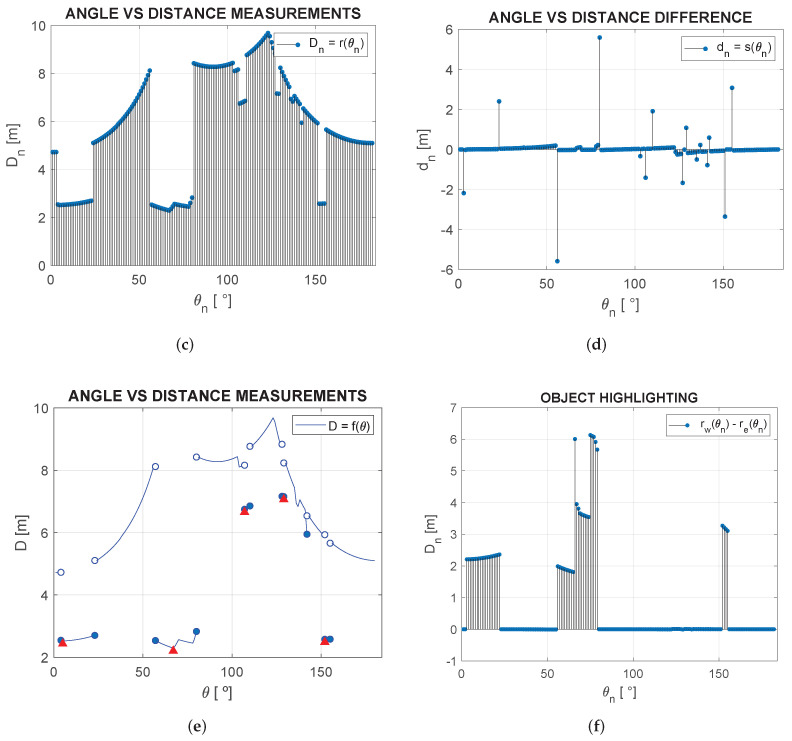
Simulated environment with different objects (**a**) and corresponding identification of objects according to our proposed framework (**b**). In addition, functions derived from LiDAR scans as defined, representing measurements from simulated semi-structured environment in (**c**–**f**).

It is essential to acknowledge that in real-world experiments, sensor noise and information losses are common challenges, typically mitigated through signal filtering processes. However, given that addressing these issues falls outside the scope of this work, we opted to demonstrate the step-by-step implementation of the object identification process in the absence of sensor noise through simulation. Figure 8 illustrates a cluttered environment created using the CoppeliaSim simulator. In other words, Figure 8a illustrates a simulated scenario, and Figure 8b shows the corresponding 2D LiDAR data. The process described earlier was applied to detect, identify, and categorize objects. Figure 8c presents a LiDAR sweep ($r(\theta_{n})$, as defined previously), allowing for an intuitive differentiation between the highest values as walls and lower readings as objects, depending on their proximity to the robot. Upon a detailed analysis and comparison of Figure 8d,e, as discussed and defined in Section 2.2, various objects were identified by setting a similar threshold difference value (as presented in Definition 3) in $s(\theta_{n})$ and observing discontinuities in $f(\theta_{n})$. Discontinuities occurring for an angle measurement where the threshold is surpassed must represent the starting point of an object. Furthermore, local minima in each set representing an object must also represent the edge closest to the scanner, marked in Figure 8e with red triangles. The objects’ readings are shown between two dark-blue filled circles, comprising $V_{1},V_{2},\ldots ,V_{11}$, thus exhibiting five fully identified objects $O_{1},O_{2},O_{3},O_{4}$, and $O_{5}$.

By comparing Figure 8a,b, one can identify the objects marked in Figure 8e in anti-clockwise order: the first and second brown prismatic boxes, the wooden ladder, the potted plant, and the smaller brown box, as seen in Figure 8a. They are, respectively, separated with color-coded bounding boxes: red for the prismatic boxes, orange for the wooden ladder, and green for the plant, according to the topology of the $F_{k}$ faces that connect each object’s edge ($V_{k}$ represented as red circles). By observing the environment using the 2D LiDAR scan, the robot can identify objects of interest in the room and understand its distance to them.

Assuming the agent has a known starting point (e.g., a recharging dock) or a map linking each LASER sweep to a certain position, it is also possible to locate objects by storing measurements of the semi-structured environment without any objects of interest to the robot—no objects that should be handled by the agent, only uninteresting objects. Then, one can highlight any new objects by taking the algebraic difference between readings before and after objects were placed—where $r_{w}$ represents measurements with the new objects and $r_{e}$ represents the original readings of the environment. This is shown in Figure 8f, where every new $V_{k}$ and $F_{k}$ are outlined, thus locating all $O_{k}$ objects of interest in the environment, while all other data are considered noise. Given these features, it is possible to match and track specific objects throughout a scene. For instance, the three brown boxes are highlighted as an example of objects of interest.

## 4. Concluding Remarks

Addressing a crucial aspect of autonomous robot decision making, the identification and localization of objects, particularly those vital for achieving specific goals, play a pivotal role in advancing robotics. The absence of a formal and standardized framework in the existing literature has posed challenges for algorithm comparison, optimization, and strategy development. This deficiency stems from the widespread use of ad hoc definitions and modeling approaches, impeding the reproducibility and advancement of results.

Our work addresses this gap by introducing a rigorous mathematical formalization applicable to a broad range of contexts involving LiDAR point-cloud data. The results presented in Section 3 demonstrate that our method can efficiently identify and separate objects in under 50 ms in semi-structured environments. Despite the necessity of setting a threshold for object detection, which may not be automatically or dynamically determined, our approach allows flexibility to tailor this parameter to the specific requirements of each application. The simplicity of our mathematical framework ensures low computational effort and efficiency, laying the foundation for creative solutions in diverse scenarios.

In conclusion, our manuscript establishes a comprehensive framework for the development and optimization of algorithms focused on autonomous object detection, localization, and matching using 2D LiDAR data. We provide essential insights into the properties of LASER scanner data and offer guidelines for feature extraction, with potential applications ranging from direct implementation for specific tasks to indirect applications in machine learning processes. Overall, we anticipate that our analytical structure will inspire the development of coherent and effective methodologies for object detection, identification, and localization in various applications.

Finally, it is noteworthy that machine learning techniques play a crucial role in object detection. Nevertheless, interpreting or comprehending the reasons behind the success or failure of a machine learning algorithm in detecting specific objects often proves challenging. In contrast, our proposed approach presents an analytical methodology for directly extracting information from LiDAR data, facilitating user comprehension of the generated output. We posit that the integration of our framework with machine learning techniques for object identification and classification holds potential benefits. Consequently, we plan to investigate the incorporation of machine learning techniques into our proposed framework for the identification and classification of objects in future work.

## Data Availability

Data are contained within the article.

## References

[1] Yang T., Li Y., Zhao C., Yao D., Chen G., Sun L., Krajnik T., Yan Z. (2022). 3D ToF LiDAR in mobile robotics: A review. arXiv.

[2] Costa P.J., Moreira N., Campos D., Gonçalves J., Lima J., Costa P.L. (2016). Localization and navigation of an omnidirectional mobile robot: The robot@ factory case study. IEEE Rev. Iberoam. Tecnol. Aprendiz..

[3] Wang J., Xu L., Li X., Quan Z. (2013). A Proposal to Compensate Platform Attitude Deviation’s Impact on Laser Point Cloud From Airborne LiDAR. IEEE Trans. Instrum. Meas..

[4] Huang Z., Zhu J., Yang L., Xue B., Wu J., Zhao Z. (2015). Accurate 3-D Position and Orientation Method for Indoor Mobile Robot Navigation Based on Photoelectric Scanning. IEEE Trans. Instrum. Meas..

[5] Schlarp J., Csencsics E., Schitter G. (2019). Optical scanning of a laser triangulation sensor for 3D imaging. IEEE Trans. Instrum. Meas..

[6] Li Y., Ruichek Y., Cappelle C. (2013). Optimal Extrinsic Calibration Between a Stereoscopic System and a LIDAR. IEEE Trans. Instrum. Meas..

[7] Raj T., Hanim Hashim F., Baseri Huddin A., Ibrahim M.F., Hussain A. (2020). A survey on LiDAR scanning mechanisms. Electronics.

[8] Krinkin K., Filatov A., yom Filatov A., Huletski A., Kartashov D. Evaluation of Modern Laser Based Indoor SLAM Algorithms. Proceedings of the 2018 22nd Conference of Open Innovations Association (FRUCT).

[9] Bresson G., Alsayed Z., Yu L., Glaser S. (2017). Simultaneous localization and mapping: A survey of current trends in autonomous driving. IEEE Trans. Intell. Veh..

[10] Gargoum S., El-Basyouny K. Automated extraction of road features using LiDAR data: A review of LiDAR applications in transportation. Proceedings of the 2017 4th International Conference on Transportation Information and Safety (ICTIS).

[11] Zamanakos G., Tsochatzidis L., Amanatiadis A., Pratikakis I. (2021). A comprehensive survey of LIDAR-based 3D object detection methods with deep learning for autonomous driving. Comput. Graph..

[12] Yahya M.A., Abdul-Rahman S., Mutalib S. Object detection for autonomous vehicle with LiDAR using deep learning. Proceedings of the 2020 IEEE 10th International Conference on system Engineering and Technology (ICSET).

[13] Weon I.S., Lee S.G., Ryu J.K. (2020). Object Recognition based interpolation with 3d lidar and vision for autonomous driving of an intelligent vehicle. IEEE Access.

[14] Konolige K., Augenbraun J., Donaldson N., Fiebig C., Shah P. A low-cost laser distance sensor. Proceedings of the 2008 IEEE International Conference on Robotics and Automation.

[15] Lu S., Zhang Y., Su J. (2017). Mobile robot for power substation inspection: A survey. IEEE/CAA J. Autom. Sin..

[16] Mertz C., Navarro-Serment L.E., MacLachlan R., Rybski P., Steinfeld A., Suppé A., Urmson C., Vandapel N., Hebert M., Thorpe C. (2013). Moving object detection with laser scanners. J. Field Robot..

[17] Azim A., Aycard O. (2012). Detection, classification and tracking of moving objects in a 3D environment. Proceedings of the 2012 IEEE Intelligent Vehicles Symposium.

[18] Lindstrom M., Eklundh J.O. Detecting and tracking moving objects from a mobile platform using a laser range scanner. Proceedings of the 2001 IEEE/RSJ International Conference on Intelligent Robots and Systems. Expanding the Societal Role of Robotics in the the Next Millennium (Cat. No. 01CH37180).

[19] Gómez J., Aycard O., Baber J. (2023). Efficient Detection and Tracking of Human Using 3D LiDAR Sensor. Sensors.

[20] Lehtomäki M., Jaakkola A., Hyyppä J., Kukko A., Kaartinen H. (2010). Detection of vertical pole-like objects in a road environment using vehicle-based laser scanning data. Remote Sens..

[21] Yang B., Dong Z., Zhao G., Dai W. (2015). Hierarchical extraction of urban objects from mobile laser scanning data. ISPRS J. Photogramm. Remote Sens..

[22] Gomes T., Matias D., Campos A., Cunha L., Roriz R. (2023). A survey on ground segmentation methods for automotive LiDAR sensors. Sensors.

[23] Nunez P., Vazquez-Martin R., del Toro J.C., Bandera A., Sandoval F. Feature extraction from laser scan data based on curvature estimation for mobile robotics. Proceedings of the 2006 IEEE International Conference on Robotics and Automation, ICRA 2006.

[24] Giri P., Kharkovsky S. (2016). Detection of Surface Crack in Concrete Using Measurement Technique With Laser Displacement Sensor. IEEE Trans. Instrum. Meas..

[25] Xiong X., Adan A., Akinci B., Huber D. (2013). Automatic creation of semantically rich 3D building models from laser scanner data. Autom. Constr..

[26] Shen S., Michael N., Kumar V. Autonomous multi-floor indoor navigation with a computationally constrained MAV. Proceedings of the 2011 IEEE International Conference on Robotics and Automation.

[27] Biswas J., Veloso M. Depth camera based indoor mobile robot localization and navigation. Proceedings of the 2012 IEEE International Conference on Robotics and Automation.

[28] Wakita S., Nakamura T., Hachiya H. Laser Variational Autoencoder for Map Construction and Self-Localization. Proceedings of the 2018 IEEE International Conference on Systems, Man, and Cybernetics (SMC).

[29] Oria-Aguilera H., Alvarez-Perez H., Garcia-Garcia D. Mobile LiDAR Scanner for the Generation of 3D Georeferenced Point Clouds. Proceedings of the 2018 IEEE International Conference on Automation/XXIII Congress of the Chilean Association of Automatic Control (ICA-ACCA).

[30] Clotet E., Palacín J. (2023). SLAMICP Library: Accelerating Obstacle Detection in Mobile Robot Navigation via Outlier Monitoring following ICP Localization. Sensors.

[31] Colaço A.F., Trevisan R.G., Molin J.P., Rosell-Polo J.R., Escolà A. (2017). Orange tree canopy volume estimation by manual and LiDAR-based methods. Adv. Anim. Biosci..

[32] Andújar D., Escolà A., Rosell-Polo J.R., Sanz R., Rueda-Ayala V., Fernández-Quintanilla C., Ribeiro A., Dorado J. (2016). A LiDAR-based system to assess poplar biomass. Gesunde Pflanz..

[33] Bargoti S., Underwood J.P., Nieto J.I., Sukkarieh S. (2015). A pipeline for trunk detection in trellis structured apple orchards. J. Field Robot..

[34] Andújar D., Rueda-Ayala V., Moreno H., Rosell-Polo J., Escolà A., Valero C., Gerhards R., Fernandez-Quintanilla C., Dorado J., Griepentrog H.W. (2013). Discriminating Crop, Weeds and Soil Surface with a Terrestrial LIDAR Sensor. Sensors.

[35] Akin H.L., Ito N., Jacoff A., Kleiner A., Pellenz J., Visser A. (2013). Robocup rescue robot and simulation leagues. AI Mag..

[36] De Azevedo A.M.C., Oliveira A.S., Gomes I.S., Marim Y.V.R., da Cunha M.P.C.P., Cássio H., Oliveira G., Martins F.N. An Omnidirectional Robot for the RoboCup Junior Rescue B Competition. Proceedings of the WEROB—RoboCupJunior Workshop on Educational Robotics.

[37] Wang Y., Wang W., Liu J., Chen T., Wang S., Yu B., Qin X. (2023). Framework for geometric information extraction and digital modeling from LiDAR data of road scenarios. Remote Sens..

[38] Axelsson P. (1999). Processing of laser scanner data—algorithms and applications. ISPRS J. Photogramm. Remote Sens..

[39] Guo B., Huang X., Zhang F., Sohn G. (2015). Classification of airborne laser scanning data using JointBoost. ISPRS J. Photogramm. Remote Sens..

[40] Filin S. (2002). Surface clustering from airborne laser scanning data. Int. Arch. Photogramm. Remote Sens. Spat. Inf. Sci..

[41] Kalenjuk S., Lienhart W., Rebhan M.J. (2021). Processing of mobile laser scanning data for large-scale deformation monitoring of anchored retaining structures along highways. Comput.-Aided Civ. Infrastruct. Eng..

[42] Cheng L., Chen S., Liu X., Xu H., Wu Y., Li M., Chen Y. (2018). Registration of laser scanning point clouds: A review. Sensors.

[43] Zhou X., Wang Y., Zhu Q., Miao Z. Circular object detection in polar coordinates for 2D LIDAR data. Proceedings of the Chinese Conference on Pattern Recognition (CCPR 2016).

[44] Diosi A., Kleeman L. Laser scan matching in polar coordinates with application to SLAM. Proceedings of the 2005 IEEE/RSJ International Conference on Intelligent Robots and Systems.

[45] Wang Y., Li B., Han B., Zhang Y., Zhao W. Laser Scan Matching in Polar Coordinates Using Gaussian Process. Proceedings of the Chinese Intelligent Automation Conference.

[46] Pelenk B., Acarman T. Object detection and tracking using sensor fusion and Particle Filter. Proceedings of the 2013 IEEE International Conference on Imaging Systems and Techniques (IST).

[47] Dong H., Weng C.Y., Guo C., Yu H., Chen I.M. (2020). Real-time avoidance strategy of dynamic obstacles via half model-free detection and tracking with 2d lidar for mobile robots. IEEE/ASME Trans. Mechatron..

[48] Vaquero V., Repiso E., Sanfeliu A. (2018). Robust and Real-Time Detection and Tracking of Moving Objects with Minimum 2D LiDAR Information to Advance Autonomous Cargo Handling in Ports. Sensors.

[49] Brandão A.S., Sarcinelli-Filho M., Carelli R. (2013). An analytical approach to avoid obstacles in mobile robot navigation. Int. J. Adv. Robot. Syst..

[50] Zhang X., Lai J., Xu D., Li H., Fu M. (2020). 2D lidar-based slam and path planning for indoor rescue using mobile robots. J. Adv. Transp..

[51] Yan K., Ma B. (2020). Mapless navigation based on 2D LIDAR in complex unknown environments. Sensors.

[52] Martins F.N., Brandão A.S. (2018). Motion Control and Velocity-Based Dynamic Compensation for Mobile Robots. Applications of Mobile Robots.

